# NLRP3 Inflammasome Activation-Mediated Pyroptosis Aggravates Myocardial Ischemia/Reperfusion Injury in Diabetic Rats

**DOI:** 10.1155/2017/9743280

**Published:** 2017-09-14

**Authors:** Zhen Qiu, Shaoqing Lei, Bo Zhao, Yang Wu, Wating Su, Min Liu, Qingtao Meng, Bin Zhou, Yan Leng, Zhong-yuan Xia

**Affiliations:** Department of Anesthesiology, Renmin Hospital of Wuhan University, Wuhan, Hubei 430060, China

## Abstract

The reactive oxygen species- (ROS-) induced nod-like receptor protein-3 (NLRP3) inflammasome triggers sterile inflammatory responses and pyroptosis, which is a proinflammatory form of programmed cell death initiated by the activation of inflammatory caspases. NLRP3 inflammasome activation plays an important role in myocardial ischemia/reperfusion (MI/R) injury. Our present study investigated whether diabetes aggravated MI/R injury through NLRP3 inflammasome-mediated pyroptosis. Type 1 diabetic rat model was established by intraperitoneal injection of streptozotocin (60 mg/kg). MI/R was induced by ligating the left anterior descending artery (LAD) for 30 minutes followed by 2 h reperfusion. H9C2 cardiomyocytes were exposed to high glucose (HG, 30 mM) conditions and hypoxia/reoxygenation (H/R) stimulation. The myocardial infarct size, CK-MB, and LDH release in the diabetic rats subjected to MI/R were significantly higher than those in the nondiabetic rats, accompanied with increased NLRP3 inflammasome activation and increased pyroptosis. Inhibition of inflammasome activation with BAY11-7082 significantly decreased the MI/R injury. *In vitro* studies showed similar effects, as BAY11-7082 or the ROS scavenger N-acetylcysteine, attenuated HG and H/R-induced H9C2 cell injury. In conclusion, hyperglycaemia-induced NLRP3 inflammasome activation may be a ROS-dependent process in pyroptotic cell death, and NLRP3 inflammasome-induced pyroptosis aggravates MI/R injury in diabetic rats.

## 1. Introduction

Acute myocardial infarction (AMI) remains a major cause of morbidity and mortality among diabetic patients [1]. Ischemia/reperfusion (I/R) causes a reduction of arterial blood supply to tissues, followed by the restoration of perfusion and consequent reoxygenation [2]. Increasing evidence has shown that diabetes increases the sensitivity to myocardial ischemia/reperfusion (MI/R), and following AMI, diabetic patients had a larger infarct size and a higher new congestive heart failure rate than nondiabetic patients [3]. This may be due to diabetes-mediated metabolic disorders, including hyperglycaemia, insulin resistance, and dyslipidaemia [4]. However, the underlying mechanisms by which diabetes aggravates MI/R are not clear.

Hyperglycaemia-induced reactive oxygen species (ROS) generation and inflammatory response are involved in the severity of MI/R injury [5–7]. The increased ROS could induce the release of inflammatory-related signaling factors, such as nuclear factor-kB (NF-kB) and nod-like receptor (NLR) inflammasome [8]. In diabetes, hyperglycaemia-induced dysfunctional mitochondria result in enhanced ROS production, which may activate inflammasomes, mediators of inflammatory responses. Activation of inflammasomes can be inhibited by antioxidants, such as silent information regulator 1 (SIRT-1) [9]. Inflammation is a response of the body to tissue injury and plays an essential role in tissue repair [10, 11]. Diverse stimuli, including pathogen-associated molecular patterns (PAMPs) and damage-associated molecular patterns (DAMPs), can activate inflammasomes to mediate the production of proinflammatory cytokines, such as mature interleukin-1*β* (IL-1*β*) and IL-18 [12–14]. NLRs (NLRP1, NLRP3, NLRC4, and NLRC5), pyrin and HIN domaintein (PYRIN), and absent in melanoma 2 (AIM2) are the major members of the inflammasome family [15]. Inflammasomes are protein complexes that include nucleotide-binding oligomerization domain (NACHT), apoptosis-associated speck-like protein (ASC), and procaspase-1 protein [16]. The NLR protein-3 (NLRP3) inflammasome is currently the best characterized inflammasome and consists of NLRP3, ASC, and procaspase-1, and NLRP3 inflammasome can activate procaspase-1 cleaves into p20 and p10 subunits that form the active caspase-1, which then leads to maturation and secretion of IL-1*β* and IL-18 [17]. A variety of structurally dissimilar agonists, including pathogens, extracellular ATP, urate crystallisation, virus-associated DNA, RNA, pore-forming toxins, and DAMPs (K^+^ efflux, lysosomal destabilization, and mitochondrial ROS), can activate the NLRP3 inflammasome to mediate downstream inflammatory responses [18, 19].

Inflammasome-mediated caspase-1 activation results in a proinflammatory form of programmed cell death known as pyroptosis. Pyroptosis is a recently identified type of programmed cell death. Caspase-1-dependent pyroptosis was first reported in mouse macrophages infected with the Gram-negative bacteria *Shigella flexneri* [20]. As a protease, caspase-1 has a basic function processing the inactive precursors of IL-1*β* into mature inflammatory cytokines; thus, it was called IL-1*β*-converting enzyme [21]. Multiple studies have confirmed that caspase-1-mediated pyroptosis is extensively involved in bacterial diseases and inflammatory processes [22]. In recent years, pyroptosis has been shown to contribute to the development of infectious diseases, nervous system-related diseases, atherosclerosis, and other diseases [23–25]. NLRP3 inflammasome-activated NLRP3/ASC-dependent inflammatory responses result in the release of significant amounts of caspase-1 and IL-1*β*, and these innate immune responses play an important role in diabetic cardiomyopathy, myocardial infarction, and MI/R injury [26–28]. Previous reports have indicated that the executor caspase of pyroptosis (activated caspase-1), which is induced by the NLRP3 inflammasome, is increased in a diabetic rat model and plays an important role in the development of diabetic cardiomyopathy [24, 29, 30]. However, the role of pyroptosis induced by ROS-mediated NLRP3 inflammasome activation in hyperglycaemia or hypoxia-induced cardiomyocyte death in diabetic status and whether diabetes-aggravated MI/R injury is related to pyroptosis are unclear. Recent studies indicated that the ROS-mediated NLRP3 inflammasome activation pathway in cardiovascular endothelial cells induced MI/R injury [31]. ROS mediate hepatocellular NLRP3 inflammasome activation, inflammatory responses, and lipid accumulation, providing a novel mechanism for fructose-induced nonalcoholic fatty liver disease pathogenesis [32]. Therefore, we hypothesized that pyroptosis induced by ROS-mediated NLRP3 inflammasome activation plays a fundamental role in the severity of MI/R injury in diabetes.

## 2. Materials and Methods

### 2.1. Animals and Experimental Groups

Healthy male adult Sprague-Dawley rats weighing 210–230 g, 6–8 weeks of age, were provided by Beijing HFK Bioscience Co. Ltd. All rats were housed in the Animal Centre of Renmin Hospital of Wuhan University in a standard environment, and experimental protocols were approved by the Bioethics Committee of Renmin Hospital of Wuhan University. All animals fasted 12 h, and the rats were administered a single intraperitoneal injection of 60 mg/kg streptozotocin (STZ) (Sigma, USA) dissolved in citrate buffer to induce diabetes as described previously [33]. As a control, normal rats were administered the same amount of citrate buffer alone. After 72 h (with 6 h fasting), a fasting blood glucose level > 16.7 mmol/L indicated hyperglycaemia, and the animals showed increased consumption of food and water and increased urination, demonstrating that the diabetic rat model was successful.

*In vivo*, the MI/R injury model was established as previously described [33, 34]. Briefly, the rats were anaesthetized by intraperitoneal injection of 1% pentobarbital sodium (60 mg/kg) and then received mechanical ventilation from an animal ventilator after endotracheal intubation. Animals were assayed using a II lead electrocardiogram (ECG), with an invasive arterial puncture to measure hemodynamics. The chest was opened to expose the heart at the fourth intercostal space of the left subclavian midline, and then, I/R was induced by ligating the left anterior descending coronary artery (LAD) for 30 min followed by reperfusion for 2 h. The sham groups underwent the same surgical procedures without LAD ligation. The criteria of ischemic success are as follows: the apical and anterior wall of the left ventricle became white, the ECG showed a widened QRS complex, the ST segment was elevated, height tip of the T wave was heightened, and the ventricular wall motion decreased. The criteria of reperfusion success are as follows: apex and anterior wall of the left ventricle recovered and turned red, and the ECG showed a normal ST.

After 8 weeks, both diabetic (DM) and nondiabetic control (Ctrl) rats were randomly divided into four groups: (1) Ctrl + sham (S); (2) Ctrl + I/R; (3) DM + S; and (4) DM + I/R. To determine the effect of the inflammasome inhibitor BAY11-7082 on MI/R injury in diabetic and nondiabetic rats, we performed another experiment including the following groups: (1) Ctrl + I/R; (2) Ctrl + I/R + BAY11-7082; (3) DM + I/R; and (4) DM + I/R + BAY11-7082. The inflammasome inhibitor BAY11-7082 (5 mg/kg dissolved in 1% DMSO) (Selleckchem) was administered 10 min before reperfusion by intraperitoneal injection [31].

### 2.2. Hemodynamic Assessment

The hemodynamic measurements were monitored continuously during the whole I/R. ECG monitoring and invasive arterial monitoring via right common carotid artery catheterization were used to record hemodynamic data (Bene View T5, Xi'an Jutian Medical Equipment Co. Ltd., China). The heart rate (HR), mean arterial pressure (MAP), and rate pressure product (RPP) were monitored at 5 min before ischemia (baseline), 0 min and 30 min after ischemia, and 30 min and 120 min after reperfusion.

### 2.3. Infarct Size Measurement

At the end of the reperfusion, six rats were randomly selected from each group to measure the myocardial infarct size using 0.3% Evans Blue dye (Sigma, USA) and 1% 2,3,5-triphenyltetrazolium chloride (TTC) staining (Sigma, USA). The myocardial area at risk (AAR) and infarct size were detected with a scanner (Epson, v30, Japan), and data were analyzed with an image analysis system (Image-Pro plus; Media Cybernetics) as described previously [33, 34]. The blue area was normal myocardium, red indicated ischemic myocardium, pale denoted myocardial infarction, and the infarct size and the percentage of AAR were calculated.

### 2.4. Creatine Kinase-MB (CK-MB) and Lactate Dehydrogenase (LDH) Measurement

At the end of reperfusion, we collected the arterial blood samples to measure the level of serum CK-MB using CK-MB isoenzyme (Jiancheng, Nanjing, China) assay kits according to the manufacturer's instructions. Serum LDH is a major indicator of MI/R injury, and cellular injury was determined using an LDH assay kit (Jiancheng, Nanjing, China) according to the manufacturer's instructions.

### 2.5. Transmission Electron Microscopy (TEM)

At the end of reperfusion, we collected 1 mm^3^ tissue from the left ventricle of the hearts and immediately fixed it in 2.5% glutaraldehyde for 6 h. The samples were prepared by professional teachers of the Electron Microscopy Centre of Renmin Hospital of Wuhan University. Finally, specimens were detected by TEM (TEM, HT7700, Japan).

### 2.6. Immunohistochemistry

For immunohistochemistry detection, after reperfusion, tissues from the apical heart region were collected, fixed with 4% buffered paraformaldehyde, and embedded in paraffin. Heart histology was assessed by immunohistochemistry with primary antibodies against NLRP3 or caspase-1 under an upright Metallurgical Microscope (Canon, Tokyo, Japan). At least two different sections from each specimen were examined.

### 2.7. H9C2 Cell Culture and H/R Injury

H9C2 cardiomyocytes were cultured in Dulbecco's modified Eagle's medium (DMEM) (Gibco Laboratories) supplemented with 10% fetal bovine serum (FBS) (Gibco Laboratories) and 1% penicillin/streptomycin in an atmosphere of 90% air and 10% CO_2_ at 37°C as described previously [33]. When the density of the cells reached 80–90%, we trypsinized the cells with 0.05% trypsin/1 mM EDTA (HyClone, USA) and plated them onto 6-well culture plates (10^5^ cells per well) for experimental treatments. The cells were randomly divided into the following experimental groups: (1) low glucose (LG) (5.5 mM); (2) LG + H/R; (3) HG + Ac-YVAD-CMK (50 *μ*M); (4) high glucose (HG) (30 mM); (5) HG + H/R; and (6) HG + H/R + Ac-YVAD-CMK (50 *μ*M). Ac-YVAD-CMK at a nontoxic concentration that had no effect on morphology or cell viability of H9c2 cells used to inhibit the caspase-1 activity. To explore the effect of the inflammasome inhibitor BAY11-7082 and the ROS inhibitor N-acetylcysteine (NAC, Sigma) on H/R injury in HG or LG conditions, we performed another experiment with the following groups: (1) LG + H/R; (2) LG + H/R + BAY11-7082 (5 *μ*M) [35–37]; (3) LG + H/R + NAC (10 mmol/L) [36, 38, 39]; (4) HG + H/R; (5) HG + H/R + BAY11-7082 (5 *μ*M); and (6) HG + H/R + NAC (10 mmol/L). We used these inhibitors or the vehicle DMSO at nontoxic concentrations that had no effect on morphology or cell viability of H9c2 cells. The experimental groups of cells were incubated in normal glucose medium for 24 h. When the cells reached 60–70% confluence, they were incubated in serum-free medium overnight and then exposed to HG or/and inhibitor for 24 h in minimal essential medium with 1% FBS. Subsequently, H9C2 cells were subjected to hypoxic conditions (1% O_2_/94% N_2_/5% CO_2_) for 4 h, followed by reoxygenation for 2 h.

### 2.8. Cell Viability Assay

Cell viability was determined using a CCK-8 Assay Kit (Jiancheng, Nanjing, China) in 96-well plates. After H9c2 cells were cultured and treated in 96-well plates, 10 *μ*L of CCK-8 reagent was added to each well and then incubated for 3 h in darkness. The absorbance was detected at 450 nm using a Perkin Elmer Microplate reader (PerkinElmer Victor 1420, USA). The mean optical density (OD) of each group was used to calculate the percent of cell viability with the following formula: cell viability = treatment group OD/control group OD × 100%.

### 2.9. Caspase-1 Activity Assay

The caspase-1 activity was detected using a caspase-1 activity assay kit (Beyotime, China) according to the manufacturer's instructions. This assay kit is based on the ability of caspase-1 to catalyse the substrate acetyl-Tyr-Val-Ala-Asp p-nitroanilide (Ac-YVAD pNA) to produce yellow p-nitroaniline (pNA), which has a strong absorption at 405 nm. Thus, the activity of caspase-1 can be assessed by measuring the absorbance of pNA using a standard pNA curve.

### 2.10. LDH Activity Assay

For the analysis of the extent of intracellular injury in pyroptotic cell death, LDH activity in culture medium was measured using a colorimetric assay kit (Jiancheng, Nanjing, China) according to the manufacturer's instructions. The results were compared with the total LDH released from cells.

### 2.11. Measurement of IL-1*β* Level

IL-1*β* level in cultured H9C2 cardiomyocyte supernatants was measured by using enzyme-linked immunosorbent assay (ELISA) (Jiancheng, Nanjing, China) according to the manufacturer's instructions.

### 2.12. Calcein-AM/Propidium Iodide (PI) Staining Assay

Calcein-AM/PI double staining was used to quantify living and dead cells for the cell death assay. Calcein-AM is a fluorescent staining reagent for live cells that can penetrate living cell membranes and form a film impermeable to polar molecules, such as calcein, which is retained and observed as bright-green fluorescence. PI cannot pass through the cell membrane of living cells but can cross the membrane of dead cells to reach the nucleus and embed in cellular DNA, producing red fluorescence. After stimulation, cells were mixed with 1x assay buffer and were then stained with 2 *μ*M calcein-AM and 4.5 *μ*M PI at 37°C for 30 min. The images were acquired using a fluorescence microscope (Olympus IX51). The average fluorescence intensity was assessed with Image Pro advanced software.

### 2.13. ROS Measurement

Intracellular ROS level was tested by dichloro-dihydro-fluorescein diacetate (DCFH-DA) assays (Sigma, USA). Briefly, cells were incubated with 50 *μ*M DCFH-DA at 37°C for 30 min in darkness. Then, the cells were washed twice using cold PBS. The fluorescence intensity of intracellular ROS was recorded using fluorescence microscopy (Olympus IX51). The average fluorescence intensity was analyzed using an image analysis system (ImageJ; National Institutes of Health).

### 2.14. Western Blot Analysis

Western blot analysis was performed as described previously [34] using antibodies against NLRP3 (1 : 200, Novus, NBP2-12446), ASC (1 : 200, Santa Cruz, sc-22514-R), caspase-1 (1 : 200, Santa Cruz, sc-514), IL-1*β* (1 : 1000, Abcam, ab9722), and GAPDH (1 : 1000, CST, D16H11). GAPDH was used as a loading control. The protein bands were detected with an Odyssey color infrared laser scan-imaging instrument (Li-Cor, USA).

### 2.15. Statistical Analysis

All values are expressed as the mean ± SD. Differences among experimental groups were analyzed by one-way ANOVA or two-way ANOVA followed by a Bonferroni post hoc test. *P* values < 0.05 were considered to be statistically significant. Statistical tests were performed using GraphPad Prism version 6.0 (GraphPad Software, USA).

## 3. Results

### 3.1. Diabetic Rats Were More Susceptible to MI/R Injury Than Nondiabetic Rats

As shown in Table 1, after STZ injection, the diabetic rats had notable diabetic symptoms, such as hyperglycaemia and weight loss. The body weight of the diabetic rats decreased, but their plasma glucose level increased compared to that of nondiabetic rats (Table 1).

**Figure 1:**
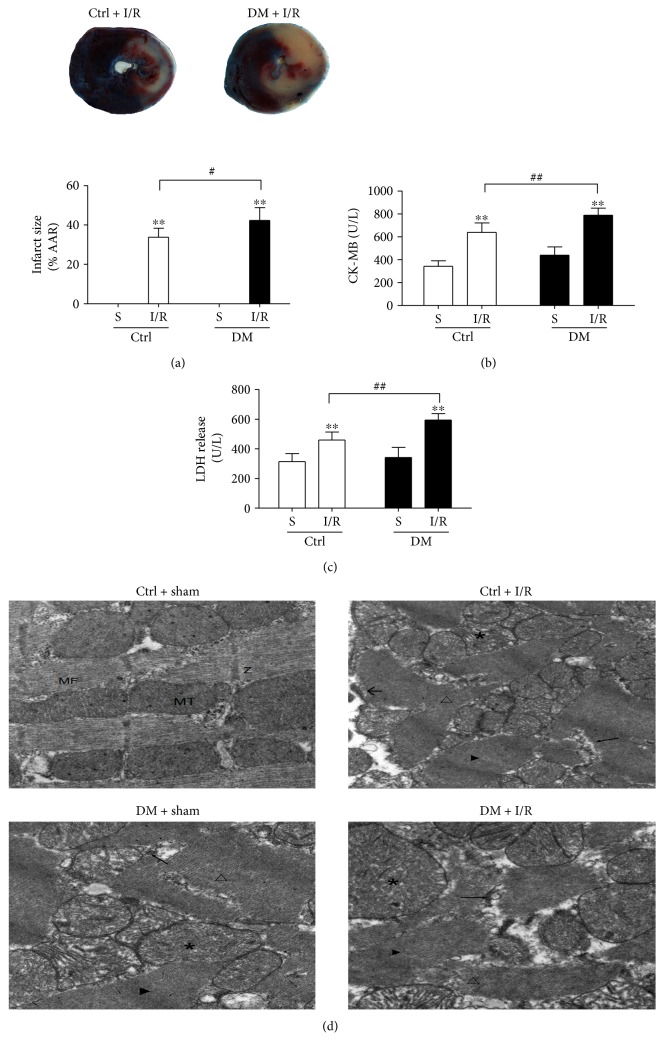
Diabetes aggravated the degree of MI/R injury in rats. The infarct size was detected by TTC staining (a). The levels of CK-MB and LDH release were determined by enzyme activity assay kits ((b) and (c)). The ultrastructural changes of rat hearts were detected by TEM (d): normal myofibrils (MF); normal mitochondria (MT); normal Z-lines (Z); disorganized myofibrils (△); swollen mitochondria (∗); expanded sarcoplasmic reticulum (←); disappearance of the Z-line (▶); and dissolved muscle cell membrane (←). Data are expressed as the mean ± SD. *n* = 6. ^∗∗^*P* < 0.01 versus sham; ^#^*P* < 0.05 and ^##^*P* < 0.01 versus Ctrl + IR.

After 8 weeks, both nondiabetic and diabetic rats subjected to MI/R injury showed higher infarct size (Figure 1(a)) than sham-operated rats, as well as increased levels of CK-MB and LDH (Figure 1(b) and Figure 1(c)). Meanwhile, the hemodynamic measurements in the Ctrl group and DM group rats were substantially altered after MI/R. After ischemia, HR, MAP, and RPP were significantly decreased and were further decreased after reperfusion as shown in Table 2. Compared with the Ctrl + I/R group, the HR, MAP, and RPP of diabetic rats were decreased following MI/R (Table 2). Interestingly, following I/R stimulation, the infarct size (Figure 1(a)), CK-MB (Figure 1(b)), and LDH release (Figure 1(a)) in diabetic rats were substantially increased compared with those in nondiabetic rats. We next confirmed MI/R injury by TEM. As shown in Figure 1(d), compared with the control rats, the diabetic rats exhibited severe damage of the left ventricular ultrastructure and interstitial cardiac fibrosis. Meanwhile, the diabetic rats subjected to MI/R showed more severe myofibril dysfunction, swollen mitochondria, and sarcoplasmic reticulum expansion compared to control rats subjected to MI/R (Figure 1(d)). These results indicated that the MI/R injury was significantly exacerbated in diabetic rats compared with that in nondiabetic rats.

### 3.2. The Exacerbated MI/R Injury in Diabetic Rats Was Associated with Increased Activation of the NLRP3 Inflammasome and Upregulated Expression of Caspase-1 and IL-1*β*

**Figure 2:**
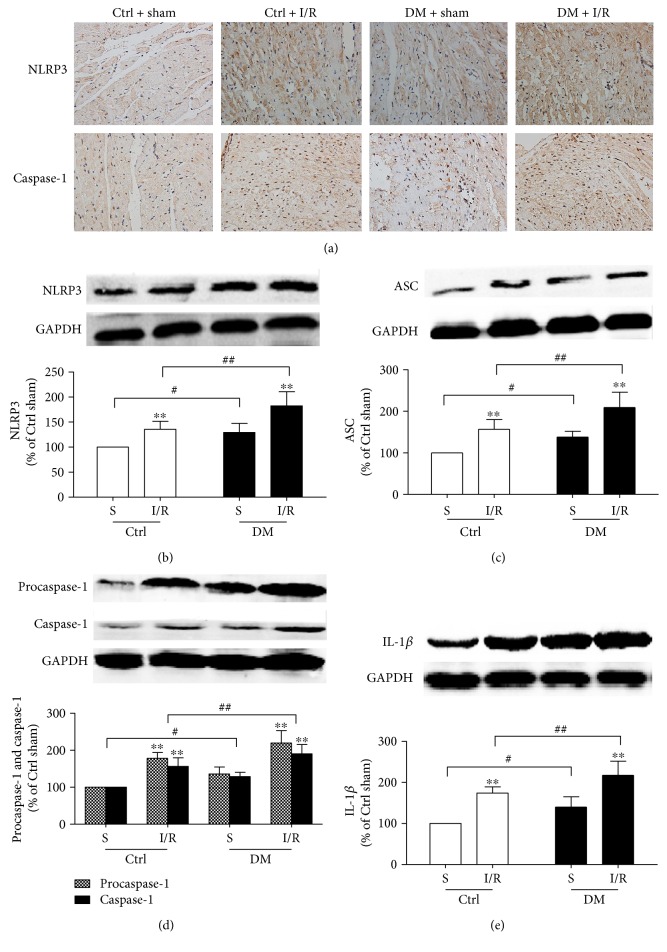
The activation of the NLRP3 inflammasome and expression of caspase-1 and IL-1*β* were increased in diabetic rats after MI/R insult. NLRP3 and caspase-1 expression in heart tissues were examined by immunohistochemistry (a). The expression of NLRP3 (b), ASC (c), procaspase-1 and caspase-1 (d), and IL-1*β* (e) were analyzed by Western blot. Data are expressed as the mean ± SD. *n* = 8. ^∗∗^*P* < 0.01 versus sham; ^#^*P* < 0.05 versus Ctrl + sham; and *^##^P* < 0.01 versus Ctrl + I/R.

The NLRP3 inflammasome has been reported to play a novel role in MI/R injury and also participates in pyroptosis [26, 40]. Activated caspase-1 indicates the presence of pyroptosis, and IL-1*β* is an early mediator for the proinflammatory response in I/R injury [13]. We therefore measured NLRP3 inflammasome, caspase-1, and IL-1*β* expression in diabetic rats subjected to MI/R injury. As shown in Figure 2, compared with the nondiabetic rats, the diabetic rats exhibited increased level of the NLRP3 inflammasome, ASC, and procaspase-1 and higher expression of activated caspase-1 (p10) and IL-1*β* proteins. When the rats were subjected to MI/R, NLRP3 (Figure 2(a) and Figure 2(b)), ASC (Figure 2(c)), and procaspase-1 (Figure 2(a) and Figure 2(d)) were further increased, as well as activated caspase-1 (Figure 2(d)) and mature IL-1*β* (Figure 2(e)), in both the diabetic and nondiabetic groups. Additionally, compared to the nondiabetic rats, diabetic rats showed significant increases in NLRP3, ASC, procaspase-1, activated caspase-1, and IL-1*β* following MI/R (Figure 2). These results indicated that the levels of the NLRP3 inflammasome, activated caspase-1, and the inflammatory cytokine IL-1*β* were increased in diabetic conditions. Following MI/R insult, activation of the NLRP3 inflammasome was increased to induce the activity of caspase-1 and the release of IL-1*β*, which mediated pyroptosis to exacerbate the MI/R injury, in diabetic rats compared with that in nondiabetic rats.

### 3.3. The Inflammasome Inhibitor BAY11-7082 Attenuated MI/R Injury and Decreased Pyroptotic Cell Death by Inhibiting Activation of the NLRP3 Inflammasome and Expression of Caspase-1 and IL-1*β* in Diabetic Rats

**Figure 3:**
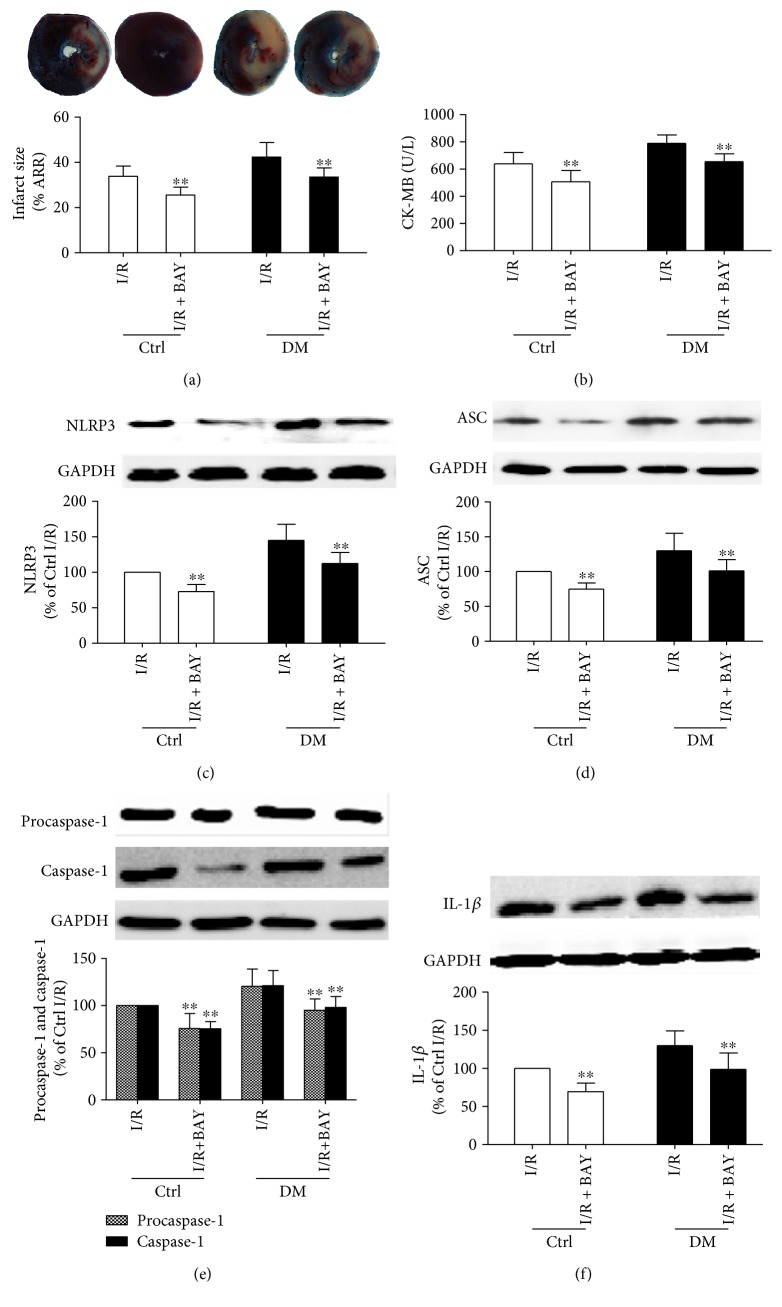
The inflammasome inhibitor reduced the myocardial infarct size and decreased activation of the NLRP3 inflammasome, IL-1*β*, and caspase-1 (p10) in nondiabetic and diabetic rats after MI/R. The infarct size was detected by TTC staining (a). The CK-MB activities were determined by enzyme activity assay kits (b). The expression of NLRP3 (c), ASC (d), procaspase-1 and caspase-1 (e), and IL-1*β* (f) was analyzed by Western blot as shown. Data are expressed as the mean ± SD. *n* = 6 to 8. ^∗∗^*P* < 0.01 versus IR.

To determine whether downregulation of NLRP3 reduces the MI/R injury, we next treated the rats with the inflammasome inhibitor BAY11-7082. As shown in Figure 3, BAY11-7028 reduced the myocardial infract size (Figure 3(a)) and significantly decreased the levels of CK-MB (Figure 3(b)) in control and diabetic rats subjected to MI/R. As shown in Table 2, the HR, MAP, and RPP were increased in BAY11-7082-treated groups compared with the non-BAY11-7082 groups. Briefly, there was a significant difference in the RPP between the Ctrl + I/R and Ctrl + I/R + BAY11-7082 groups. Compared with those in the DM + I/R group, the HR and RPP were significantly decreased in the DM + I/R + BAY11-7082 group. In addition, the inflammasome BAY11-7082 inhibitor significantly inhibited NLRP3 inflammasome activation in diabetic rats with MI/R as shown by the decreased expression of NLRP3 (Figure 3(c)), ASC (Figure 3(d)), and procaspase-1 (Figure 3(e)). The expressions of caspase-1 p10 (Figure 3(e)) and IL-1*β* production (Figure 3(f)) were also substantially decreased by the inflammasome inhibitor. These results indicated that BAY11-7082 could ameliorate MI/R injury by reducing the NLRP3 inflammasome activation and inhibiting caspase-1-dependent pyroptosis and inflammatory reactions in diabetic rats.

### 3.4. H/R Stimulated NLRP3 Inflammasome Activation to Induce Cell Injury and Pyroptosis in Cultured H9C2 Cells in HG Conditions

**Figure 4:**
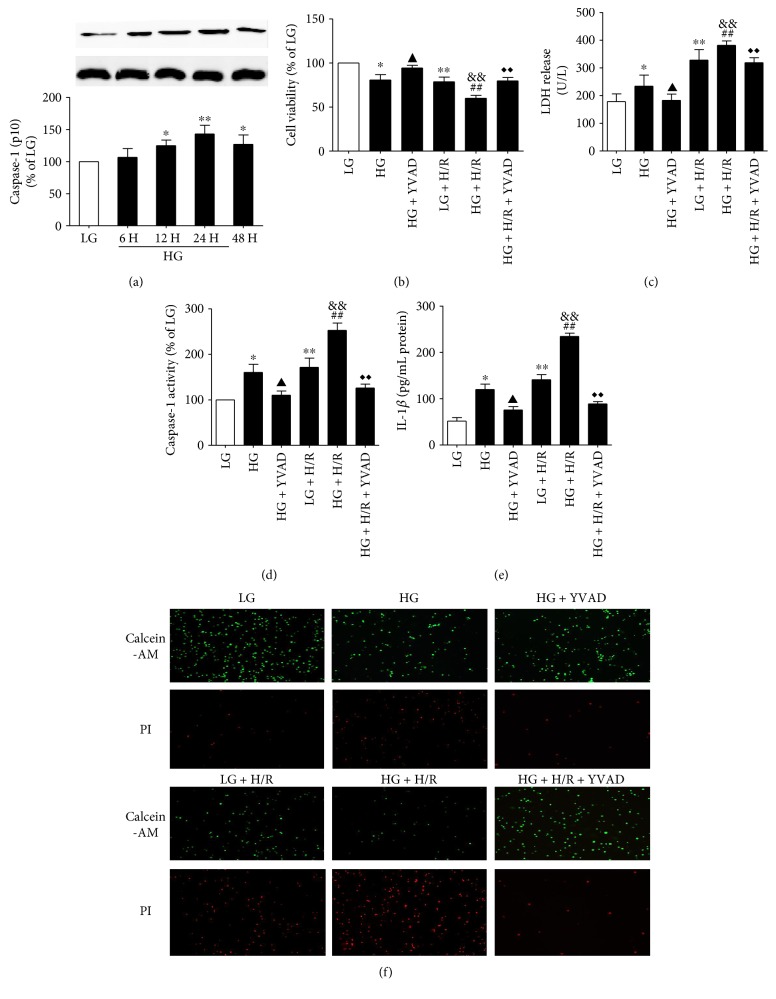
HG and H/R stimulate caspase-1 activation and pyroptosis in cultured H9C2 cells. The expression of caspase-1 (p10) was analyzed by Western blot (a). Cell viability was detected by CCK-8 assay (b). The LDH release was detected by activity assays (c). Caspase-1 activity was determined by caspase-1 activity assay (d). IL-1*β* content was measured by using ELISA kits (e). Calcein-AM/PI staining was used for the cell death assay; green indicates living cells, and red indicates dead cells (f). Data are expressed as the mean ± SD. *n* = 5 per group. ^∗^*P* < 0.05 and ^∗∗^*P* < 0.01 versus LG; ^▲^*P* < 0.01 and ^&&^*P* < 0.01 versus HG; ^##^*P* < 0.01 versus LG + H/R; and ^◆◆^*P* < 0.01 versus HG + H/R.

Compared with the LG group, HG significantly increased the activation of caspase-1 (Figure 4(a)) in cultured H9C2 cells from 6 h to 48 h. After 24 h of exposure to HG, the activation of caspase-1 peaked compared with that of the LG control. Thus, we chose 24 h as the HG stimulation time in the subsequent experiments.

As shown in Figure 4(b), compared with the LG group, the HG group showed significantly decreased cell viability, which was further reduced by H/R stimulation. The LDH release (Figure 4(c)), caspase-1 activity (Figure 4(d)), and cell supernatant IL-1*β* (Figure 4(e)) level were increased in HG and significantly increased by H/R stimulation. However, when the caspase-1 inhibitor Ac-YVAD-CMK was used, the cell viability was significantly increased and reversed the cell injury induced by HG and H/R. In addition, the HG and H/R-increased LDH level, caspase-1 activity, and IL-1*β* level were significantly reversed by the caspase-1 inhibitor Ac-YVAD-CMK. We next measured caspase-1 activity using a caspase-1 activity assay kit and pyroptosis by calcein-AM/PI staining, which was used to test the form of cell death especially as the characteristic detection method of pyroptosis in the living and dead cells [41]. For pyroptosis by calcein-AM/PI staining, as shown in Figure 4(f), HG and H/R significantly induced pyroptotic cell death compared with LG conditions and further increased by H/R stimulation. Treatment with caspase-1 inhibitor Ac-YVAD-CMK evidently suppressed the pyroptotic cell death.

**Figure 5:**
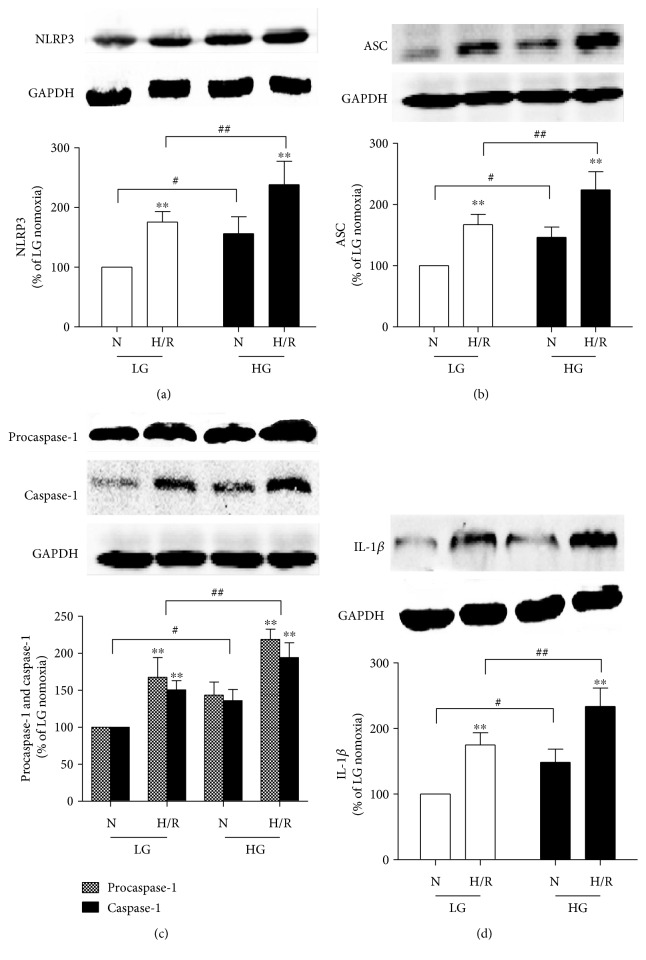
Protein expression in HG and H/R conditions. Western blotting for NLRP3 (a), ASC (b), procaspase-1 and caspase-1 (p10) (c), and IL-1*β* (d) in H9C2 cells. Data are expressed as the mean ± SD. *n* = 5 per group. ^∗∗^*P* < 0.01 versus N; ^#^*P* < 0.01 versus LG; and ^##^*P* < 0.01 versus LG + H/R.

To evaluate the roles of the NLRP3 inflammasome and pyroptosis in cells exposed to HG and H/R stimulation, we next measured the NLRP3 inflammasome and pyroptosis-related proteins. The results demonstrated that HG conditions could significantly increase the expression of NLRP3 (Figure 5(a)), ASC (Figure 5(b)), procaspase-1 and caspase-1 (Figure 5(c)), and IL-1*β* (Figure 5(d)), and these alterations were further increased by H/R stimulation. Activation of the NLRP3 inflammasome was further increased in H9C2 cells in HG and H/R conditions compared with that in LG and H/R conditions. Increases in pyroptotic cell death proteins were detected in H9C2 cells in HG and H/R conditions as shown by the increased caspase-1 activity and IL-1*β* level. All these results demonstrated that NLRP3 inflammasome-induced pyroptosis and inflammatory reactions were activated in cultured H9C2 cells by H/R stimulation and further increased by HG conditions.

### 3.5. The Inflammasome Inhibitor Attenuated Cell Injury and Cell Pyroptotic Death in Cultured H9C2 Cells Exposed to HG and H/R Conditions by Decreasing the Activation of the NLRP3 Inflammasome, Activated Caspase-1, and IL-1*β*

**Figure 6:**
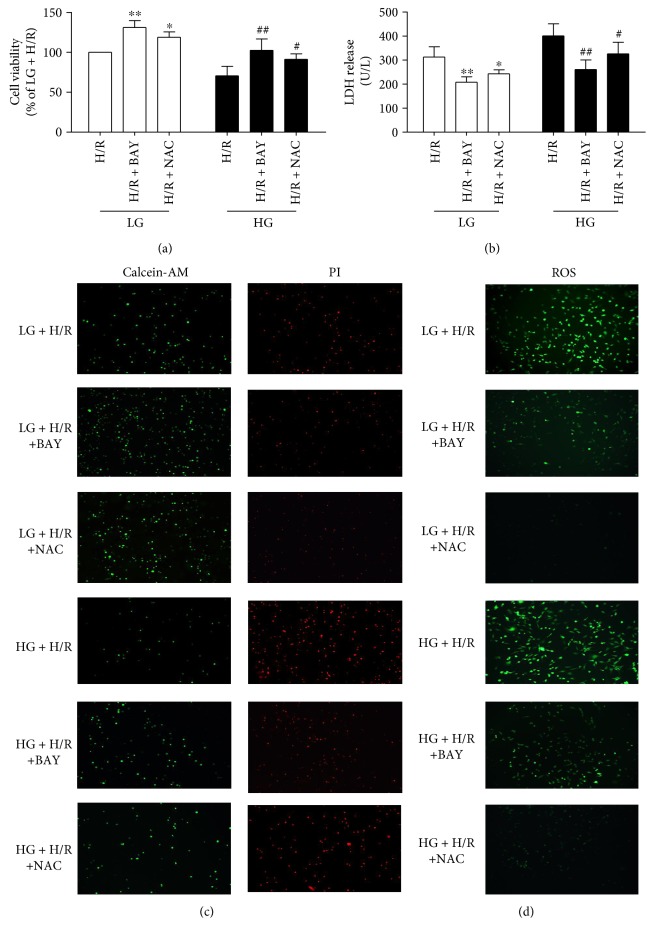
The inflammasome inhibitor and ROS scavenger NAC reduced the inflammasome levels and cell death induced by HG and H/R conditions. The cell viability (a) and the LDH release (b) were assessed. Calcein-AM/PI was used to detect pyroptosis (c). DCFH-DA assay was used to assess cellular ROS production (d). Data are expressed as the mean ± SD. *n* = 5 per group. ^∗^*P* < 0.05 and ^∗∗^*P* < 0.01 versus LG + H/R; ^#^*P* < 0.05 and ^##^*P* < 0.01 versus HG + H/R.

To investigate the effects of NLRP3 inflammasome on HG and H/R-induced pyroptotic cell death in H9C2 cells, we treated the cells with the inflammasome inhibitor BAY11-7082. As shown in Figure 6(a) and Figure 6(b), BAY11-7082 significantly increased cell viability and decreased LDH release in H9C2 cells exposed to HG and H/R insult and attenuated pyroptotic cell death (Figure 6(c)). As shown in Figure 6(d), ROS production was significantly decreased by the BAY11-7082 inhibitor in cultured H9C2 cells exposed to H/R insult.

**Figure 7:**
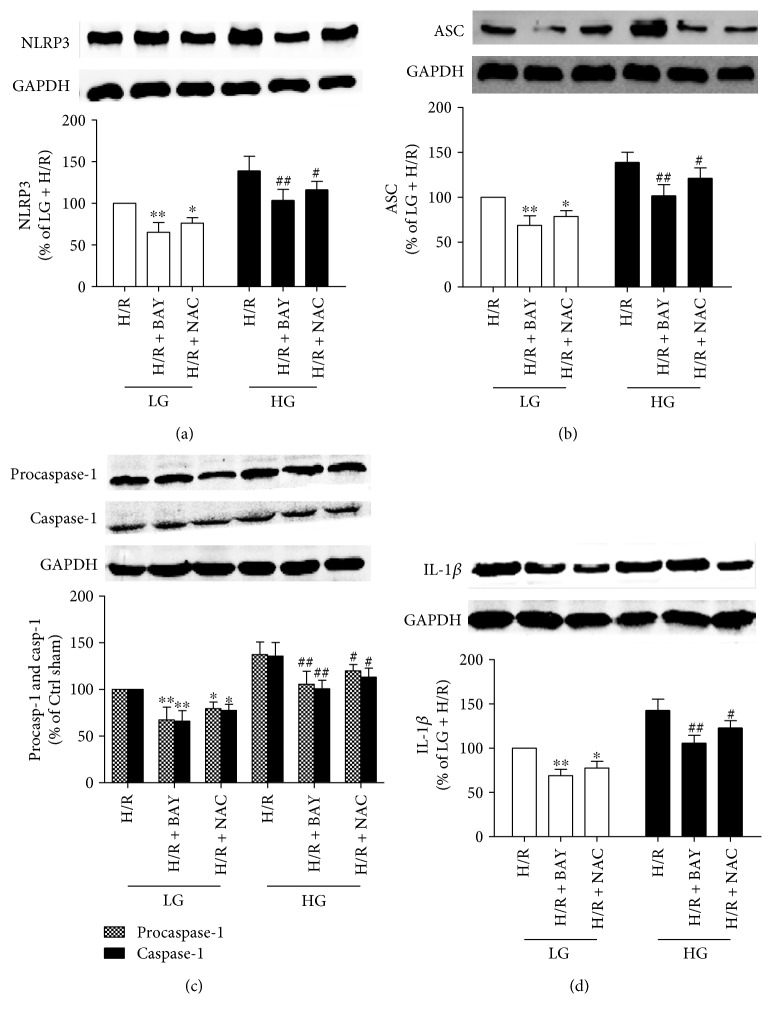
The ROS scavenger inhibited the activation of NLRP3 inflammasomes in H9C2 cells exposed to H/R injury. The expression levels of NLRP3 (a), ASC (b), procaspase-1 and caspase-1 (p10) (c), and IL-1*β* (d) were detected by Western blot. Data are expressed as the mean ± SD. *n* = 5 per group. ^∗^*P* < 0.05 and ^∗∗^*P* < 0.01 versus LG + H/R; ^#^*P* < 0.05 and ^##^*P* < 0.01 versus HG + H/R.

As shown in Figure 7, the inflammasome inhibitor significantly inhibited NLRP3 inflammasome activation and the expression of executors of pyroptosis compared to that of the control groups (I/R without inhibitor). The decreased NLRP3 inflammasome activation was measured by decreased expression of NLRP3 (Figure 7(a)), ASC (Figure 7(b)), and procaspase-1 (Figure 7(c)). The levels of caspase-1 (Figure 7(c)) and mature IL-1*β* (Figure 7(d)) were also significantly downregulated in the inflammasome inhibitor-treated groups. These results showed that inhibition of the NLRP3 inflammasome by BAY11-7082 inhibitor can alleviate H/R-induced pyroptotic cell injury by decreasing NLRP3 expression and NLRP3 inflammasome activation and leading to reduced caspase-1 and IL-1*β* in both LG and HG conditions.

### 3.6. ROS Mediated Cell Injury and Cell Pyroptotic Death in Cultured H9C2 Cells Exposed to HG and H/R Conditions via NLRP3 Inflammasome Activation, and the ROS Scavenger NAC Ameliorated HG and H/R-Induced Cell Injury by Inhibiting NLRP3 Inflammasome Activation and Decreasing Pyroptosis

ROS have been reported to play an important role in MI/R injury by triggering inflammatory reactions. To investigate the role of ROS in NLRP3 activation and cell death induced by H/R with or without HG, we used the ROS scavenger NAC to inhibit the production of ROS in H9C2 cells. Cell injury was significantly reversed by NAC treatment in cultured H9C2 cells exposed to H/R stimulation, as evidenced by decreased LDH activity, increased cell viability (Figure 6(a) and Figure 6(b)) and reduced pyroptosis (Figure 6(c)). As shown in Figure 6(d), ROS production was significantly increased by H/R stimulation in both LG and HG conditions. Meanwhile, NAC treatment significantly decreased the production of ROS to alleviate the pyroptotic cell death induced by HG and H/R. These results showed that inhibited production of ROS could alleviate the H/R-induced cell injury in both LG and HG conditions.

Moreover, the ROS scavenger NAC inhibited NLRP3 inflammasome activation, indicated by the decreased expression of NLRP3 (Figure 7(a)), ASC (Figure 7(b)), and procaspase-1 (Figure 7(c)) induced by HG and H/R (Figure 7). Caspase-1 (Figure 7(c)) and mature IL-1*β* (Figure 7(d)) expressions were significantly downregulated in the NAC inhibitor-treated groups. These results showed that the ROS scavenger NAC can alleviate H/R-induced cell injury by decreasing NLRP3 inflammasome activation, reducing activated caspase-1-induced pyroptotic cell death and IL-1*β* levels in both LG and HG conditions.

## 4. Discussion

In the present study, we first demonstrated that pyroptosis induced by ROS-mediated NLRP3 inflammasome activation plays an important role in inflammatory responses and MI/R injury in diabetic rats, and inhibition of NLRP3 inflammasome activation decreased MI/R injury in diabetic rats and H/R injury in HG condition H9C2 cells. Our results indicated that both the activation of the NLRP3 inflammasome, including NLRP3, ASC, and procaspase-1, and pyroptosis-related factors as activated caspase-1 (p10) and IL-1*β* were increased in diabetic rats. Moreover, when the diabetic rats underwent MI/R, NLRP3 inflammasome, activated caspase-1, and IL-1*β* showed further increases along with increased infarct size and exacerbated injury. These results suggested that the NLRP3-induced proinflammatory programmed cell death is an initial response in MI/R injury in diabetes. The inflammasome inhibitor BAY11-7082 attenuated the infarct size and injury in diabetes and nondiabetes after MI/R by decreasing NLRP3 inflammatory activation, as evidenced by decreased expression of the NLRP3 inflammasome (NLRP3, procaspase-1, and ASC protein complexes), and downregulated the expression of caspase-1 (p10) and cytokine IL-1*β*. *In vitro*, NLRP3 inflammasome activation was increased in HG conditions and after H/R stimulation. Following the H/R insult, pyroptotic cell death was aggravated in HG conditions, consistent with the higher expression of NLRP3, ASC, and procaspase-1 and increased activation of caspase-1 and IL-1*β* compared with those of control glucose conditions. The pyroptotic cell injury induced by HG and H/R was relieved by caspase-1 inhibitor AC-YVAD-CMK. Similar to *in vivo* experiments, H9C2 cells underwent H/R and were treated with the inflammasome inhibitor BAY11-7082 and NAC, which inhibited the production of ROS and blocked activation of the NLRP3 inflammasome to ameliorate the H/R injury in LG and HG conditions. These results suggest that NLRP3 inflammasome activation and NLRP3-induced caspase-1-dependent pyroptotic cell death and inflammatory responses are novel mechanisms of MI/R injury in diabetes and indicate that NLRP3 inflammasome-induced pyroptosis is a potential novel therapeutic target for MI/R injury in diabetes.

Clinical and experimental studies have demonstrated that diabetic hearts are more sensitive to I/R injury [42, 43]. Diabetes mellitus can exacerbate MI/R injury and is resistant to various therapeutic methods [44]. In diabetes, enhanced oxidative stress induced by hyperglycaemia, hyperlipidaemia, hyperinsulinaemia, and insulin resistance contributes to mitochondrial dysfunction and leads to excessive cytokine generation in the diabetic myocardium; thus, diabetes cannot readily adapt to MI/R. Diabetes can lead to excessive production of ROS, and when MI/R occurs, ion channel opening (such as K^+^ efflux) increases the production of ROS to induce inflammatory cascades. In our present study, we used STZ to generate type 1 diabetic animal models. At 8 weeks, we found that the myocardium pathological damage accompanied by inflammatory responses was increased in diabetic rats compared with that of nondiabetic rats. After MI/R, the diabetic rats showed exacerbation of MI/R injury and more severe impairment of myocardial function compared with the nondiabetic rats, along with increased myocardial infarct size and higher levels of LDH and CK-MB. These findings emphasized that diabetes can aggravate MI/R injury, consistent with the results of previous studies [34, 35, 43].

MI/R injury is characterized by increased cytokines, chemokines, and excessive leukocytes in the damaged myocardial region [45]. Studies have confirmed that during diabetic MI/R, excess oxidative stress can induce apoptosis, necrosis, and inflammatory reactions in cardiac cells as the mechanisms of MI/R injury [46–48]. When MI/R occurs, activated damage signaling pathways promote cell survival by diminishing injury and substantially inhibiting leukocytes or inflammatory mediators to reduce MI/R injury [49]. Therefore, inflammation plays a critical role in the pathophysiology of MI/R injury. As the mediator for inflammatory responses, the NLRP3 inflammasome is the most extensively characterized inflammasome and has been associated with many nonbiological danger signals, including glucose, ROS, and crystallisation, which induce a nonbacterial inflammatory response [28, 50, 51]. These proinflammatory cytokines activate an inflammatory cascade that results in the recruitment of innate immune cells and can also determine the character of the subsequent adaptive immune response. NLRP3 is characterized by its N-terminal PYD, which recruits the adaptor molecule ASC through PYD-PYD interactions, facilitating the recruitment of procaspase-1 to form the NLRP3 inflammasome complex [52]. NLRP3 inflammasomes were found to cleave the procaspase-1 to active caspase-1, which then led to processing and maturation of the inflammatory cytokine IL-1*β*. IL-1*β* is an early mediator of MI/R injury and an inflammasome-associated and pyroptosis-related signaling molecule. In the present study, we found that diabetic state increased NLRP3 inflammasome activation and further increased the expression of caspase-1 and IL-1*β* production. When the diabetic rats experienced MI/R, we discovered that both the NLRP3 inflammasome activation and pyroptosis-related signaling molecules, including caspase-1 and IL-1*β*, were substantially increased compared with that in nondiabetic rats. In addition, the *in vitro* study showed similarly increased activation of the NLRP3 inflammasome, caspase-1, and IL-1*β* in H9C2 cells in HG conditions and aggravated H/R injury. These results provided further evidence that the inflammatory reaction mediated by the NLRP3 inflammasome plays a key role in the pathogenesis of MI/R injury in diabetic rats and that caspase-1 and mature IL-1*β* activated by the NLRP3 inflammasome are important signaling pathways for MI/R injury.

Necrosis, apoptosis, and other programmed cell death pathways are involved in a variety of diseases. Recent studies have shown that there are many other forms of cell death, including pyroptosis. Pyroptosis is a proinflammatory form of lytic cell death and is initiated by the activation of inflammatory caspases, as a critical mechanism for the host response to pathogens and endogenous injury [53]. In contrast to other cell death methods, pyroptosis has unique features, including programming by inflammatory caspase activation-active caspase-1, membrane pore formation, and leakage of intracellular contents [53, 54]. Pyroptosis can result in inflammatory cytokine release and shares some features with apoptosis (including DNA fragmentation and nuclear condensation) and necrosis (such as loss of plasma membrane integrity and release of intracellular contents, such as LDH) [55]. Caspase-1 is activated by large multiprotein complexes-inflammasomes, which regulate both pyroptosis and the maturation and secretion of proinflammatory IL-1*β* as a key substrate of caspase-1 [56]. Inflammasomes can induce caspase-1-independent pyroptosis in multiorgan diseases and pathological injury [7, 57, 58]. Studies have shown that pyroptosis as a new way of cell death is closely related to the development of a variety of diseases and tissue damage [53]. Studies have indicated that hyperglycaemia increased the production of ROS to activate caspase-1 and trigger the caspase-1-dependent pyroptotic cell death, suggesting that pyroptosis mediates important pathological changes in diabetic cardiomyopathy [24, 29]. In the present study, we found typical characteristics of pyroptosis in the diabetic myocardium and myocardial tissue after MI/R in diabetic models, including increased expression of activated caspase-1, IL-1*β* production following activation of the NLRP3 inflammasome, cytoplasmic swelling, and LDH release, which aggravated MI/R injury in diabetic rats. These results suggested that pyroptosis plays a significant role in the pathogenesis of diabetic myocardium and MI/R injury in diabetes. In *vitro*, detection of caspase 1 activity, cell supernatant IL-1*β* level, and calcein-AM/PI double staining was used in combination with an LDH release assay to detect and quantify pyroptosis. We used HG and H/R to stimulate H9C2 cardiomyocytes, and the results that HG and H/R significantly increased caspase 1 activity, IL-1*β* level and pyroptotic cell death with increasing LDH release further confirmed that pyroptosis is involved and aggravated H/R injury of H9C2 cardiomyocytes in HG conditions. These data suggested that the caspase-1-dependent pyroptosis induced by NLRP3 activation contributed to diabetic myocardium and aggravated MI/R injury in diabetes.

NLRP3, ASC, and procaspase-1 forming the NLRP3 inflammasome complex were found to cleave the procaspase-1 to active caspase-1, which then leaded to processing and maturation of the inflammatory cytokine IL-1*β* that induced pyroptosis. In our current study, *in vivo*, a connection between the expression of NLRP3 and activated caspase-1 in pyroptotic cell death was observed in MI/R injury of diabetic or nondiabetic rats. Diabetic rats showed more severe injury compared with nondiabetic rats following MI/R insult. These findings indicate that the caspase-1 signaling pathway-induced pyroptosis mediated by NLRP3 activation plays a significant role in MI/R injury with diabetes, which was confirmed by the *in vitro* study. We reconfirmed that NLRP3 inflammasome activation-mediated pyroptosis aggravated MI/R injury in diabetes. BAY11-7082 is an inflammasome inhibitor, and studies have reported that BAY11-7082 could well inhibit NLRP3 inflammasome activation and decrease the MI/R injury in rat MI/R injury model [31, 37]. Studies have reported that treatment with BAY11-7082 could inhibit NLRP3 inflammasome activation, which indicated that BAY11-7082 was a potent inhibitor of the NLRP3 inflammasome independent of their inhibitory effect on the NF-*κ*B pathway, and BAY11-7082 might represent an interesting approach for the management of psoriasis-like dermatitis depending on the dual inhibition of NF-*κ*B and NLRP3 [59, 60]. In our study, we used BAY-11-7082 to inhibit the NLRP3 inflammasome activation. Our results indicated that BAY-11-7082 could inhibit NLRP3 inflammasome activation to inhibit caspase-1-dependent pyroptosis and the inflammatory response, thus alleviating MI/R injury in diabetic rats and H/R injury in HG condition H9C2 cells. With the treatment of BAY11-7082, the myocardial infract size and MI/R injury were significantly decreased in rat MI/R experiments and the cell viability was significantly increased with caspase-1 activity and LDH release level significantly decreased in H9C2 cells exposed to HG and H/R insult 7 and attenuated the pyroptotic cell death. Results showed that the expression levels of NLRP3, ASC, and procaspase-1 were significantly decreased, and the expressions of caspase-1 p10 and IL-1*β* were also downregulated both in vivo and in vitro experiments. These results indicated that BAY11-7082 has been shown to inhibit NLRP3 inflammasome activation to decrease caspase-1-dependent pyroptosis that markedly attenuated MI/R injury in diabetes.

Many activators of the NLRP3 inflammasome have already been identified; studies show that generated by NLRP3 activators, ROS acts as second messengers whose signaling drives inflammasome activation [15–17]. ROS have been shown to activate the inflammasome in MI/R injury [33, 36], ROS generation has been identified as an important mechanism by which NLRP3 inflammasome is activated, and NLRP3 activation was blocked by ROS scavengers [36]. Oxidative stress-mediated NLRP3 activation results in interaction with ASC and caspase-1 to mediate pyroptosis [32]. In the present study, we found that the activation of NLRP3 inflammasome significantly increased in a ROS-dependent manner to induce pyroptotic cell death during MI/R injury in diabetes. Inhibition of the NLRP3 inflammasome by BAY11-7082 significantly suppressed NLRP3 expression and decreased the activation of the NLRP3 inflammasome. These changes reduced the expression of activated caspase-1 and IL-1*β* in diabetes with MI/R, along with a decrease in ROS production. Moreover, we next explored the relationship between ROS and the NLRP3 inflammasome activation in H/R injury *in vitro*. As a ROS scavenger, NAC was used to inhibit the production of ROS in H9C2 cells cultured in LG or HG conditions with H/R stimulation. We found that NAC inhibited the activation of NLRP3 inflammasomes to alleviate cell injury in cardiomyocytes after H/R injury in both LG and HG conditions. Our results indicated that ROS is a novel activator for NLRP3 inflammasomes, which induce caspase-1-dependent pyroptosis in diabetic MI/R injury. Moreover, caspase-1-dependent pyroptosis induced by ROS-mediated NLRP3 inflammasome activation is an important factor for the diabetes-associated increased sensitivity to MI/R and exacerbated MI/R injury.

To the best of our knowledge, our research demonstrated for the first time that ROS-mediated NLRP3 inflammasome activation induced both pyroptosis and inflammation in diabetic MI/R injury. Targeting the NLRP3 inflammasome and pyroptosis may represent a novel strategy to decrease MI/R tissue injury. However, this study has several limitations that need further exploration. This study only investigated the role of BAY11-7082, as the inhibitor of the NLRP3 inflammasome, to investigate the effects of NLRP3 inflammasome activation in MI/R injury. Thus, further studies are needed to investigate the effects of other specific inflammatory inhibitors and NLRP3 gene knockout or silencing in MI/R injury in diabetic rats to verify the specific effects of NRLP3-inflammasome. Further investigation into the upstream signaling pathways for NLRP3 inflammasome-induced pyroptosis in diabetic MI/R injury is also the next direction.

## 5. Conclusion

In summary, our present study demonstrated that pyroptotic cell death induced by ROS-mediated NLRP3 inflammasome activation via caspase-1 activation signaling pathways may play an important role in MI/R injury in diabetic rats, and an inflammasome inhibitor or ROS scavenger (NAC) can attenuate diabetic MI/R injury. Inhibition of NLRP3 inflammasome activation to reduce pyroptosis may be a new strategy to mitigate MI/R injury. Further studies exploring the specific signaling pathways of NLRP3 inflammasome mediated-pyroptotic cell death in the development of diabetic MI/R injury are needed to confirm this hypothesis.

## Figures and Tables

**Table 1:** Characteristics of control and diabetic rats after 8 weeks.

	Ctrl	DM
Body weight (g)	380.0 ± 22.0	189.5 ± 46.5^∗∗^
Glucose (mmol/L)	5.2 ± 1.4	25.9 ± 6.4^∗∗^
Heart weight (g)	1.58 ± 0.12	0.84 ± 0.09^∗∗^

Data are expressed as the mean ± SD. *n* = 8. ^∗∗^*P* < 0.01 versus Ctrl 8 weeks after STZ injection.

**Table 2:** The hemodynamic measurements after MI/R in Ctrl and DM rats.

Parameters	Group	Baseline	Is0	Is30	Rep30	Rep120
HR (bpm)	Ctrl + I/R	325.5 ± 19.8	305.2 ± 17.3^∗∗^	289.7 ± 13.1^∗∗^	289.3 ± 17.3^∗∗^	277.1 ± 10.6^∗∗^
	Ctrl + I/R + BAY	312.7 ± 16.8	277.3 ± 21.3^∗∗^	277.7 ± 13.9^∗^	283.3 ± 15.7^∗^	285.0 ± 14.6
	DM + I/R	303.5 ± 27.0^&&^	246.2 ± 22.3^∗∗^^&&^	237.3 ± 17.4^∗∗^^&&^	239.5 ± 27.7^∗∗^^&&^	250.5 ± 32.2^∗∗^^&&^
	DM + I/R + BAY	307.2 ± 8.8	252.2 ± 13.6^∗∗^	262.2 ± 8.4^∗∗^	275.7 ± 10.2^∗∗^^$$^	282.3 ± 9.4^$$^
MAP (mmHg)	Ctrl + I/R	99.7 ± 10.2	70.5 ± 10.8^∗∗^	77.7 ± 9.2^∗^	76.7 ± 9.2^∗^	73.5 ± 11.8^∗∗^
	Ctrl + I/R + BAY	87.7 ± 10.7	61.5 ± 11.0^∗∗^	65.7 ± 5.3^∗∗^	72.3 ± 6.3^∗^	76.5 ± 4.4
	DM + I/R	91.5 ± 3.7	74.5 ± 12.8^∗∗^	75 ± 5.4^∗∗^	72.3 ± 5.9^∗∗^	63.7 ± 6.9^∗∗^
	DM + I/R + BAY	92.2 ± 2.8	69.0 ± 5.3^∗∗^	75.7 ± 8.4^∗∗^	76.5 ± 6.9^∗^	81.3 ± 9.6^$^
RPP (min. mmHg 10^3^)	Ctrl + I/R	34.7 ± 5.4	24.5 ± 3.6^∗∗^	25.7 ± 4.6^∗^	25.4 ± 6.4^∗^	22.4 ± 4.3^∗∗^
	Ctrl + I/R + BAY	29.5 ± 5.5	18.3 ± 2.5^∗∗^^##^	20.0 ± 2.4^∗∗^^##^	21.7 ± 2.3^∗∗^	22.6 ± 2.6^∗^
	DM + I/R	28.9 ± 4.3&	19.8 ± 3.4^∗∗^^&^	18.8 ± 1.6^∗∗^^&&^	19.0 ± 2.3^∗∗^^&&^	17.1 ± 2.6^∗∗^
	DM + I/R + BAY	29.6 ± 1.9	18.4 ± 0.9^∗∗^	20.6 + 2.4^∗∗^	24.3 ± 2.1^∗∗^	24.0 ± 3.0^∗^^$$^

The data are expressed as the mean ± SD. *n* = 6. ^∗^*P* < 0.05 and ^∗∗^*P* < 0.01 versus respective baseline; ^##^*P* < 0.01 versus the control I/R group; ^&^*P* < 0.05 and ^&&^*P* < 0.01 versus the control I/R group; ^$^*P* < 0.05 and ^$$^*P* < 0.01 versus the DM + I/R group. Ctrl + I/R and DM + I/R: nondiabetic and diabetic rats were subjected to 30 min ischemia followed by 2 h reperfusion. Ctrl + I/R + BAY and DM + I/R + BAY: nondiabetic and diabetic rats were subjected to 30 min ischemia followed by 2 h reperfusion and were, respectively, given the inflammasome inhibitor BAY11-7082 10 min before reperfusion by intraperitoneal injection.

## References

[1] Badalzadeh R., Mokhtari B., Yavari R. (2015). Contribution of apoptosis in myocardial reperfusion injury and loss of cardioprotection in diabetes mellitus. *The Journal of Physiological Sciences*.

[2] Eltzschig H. K., Eckle T. (2011). Ischemia and reperfusion—from mechanism to translation. *Nature Medicine*.

[3] Balakumar P., Sharma N. K. (2011). Healing the diabetic heart: does myocardial preconditioning work?. *Cellular Signalling*.

[4] Przyklenk K., Maynard M., Greiner D. L., Whittaker P. (2011). Cardioprotection with postconditioning: loss of efficacy in murine models of type-2 and type-1 diabetes. *Antioxidants & Redox Signaling*.

[5] Minutoli L., Puzzolo D., Rinaldi M. (2016). ROS-mediated NLRP3 inflammasome activation in brain, heart, kidney, and testis ischemia/reperfusion injury. *Oxidative Medicine and Cellular Longevity*.

[6] Ansley D. M., Wang B. (2013). Oxidative stress and myocardial injury in the diabetic heart. *The Journal of Pathology*.

[7] Ding M., Lei J., Han H. (2015). SIRT1 protects against myocardial ischemia-reperfusion injury via activating eNOS in diabetic rats. *Cardiovascular Diabetology*.

[8] Devi T. S., Lee I., Hüttemann M., Kumar A., Nantwi K. D., Singh L. P. (2012). TXNIP links innate host defense mechanisms to oxidative stress and inflammation in retinal Muller glia under chronic hyperglycemia: implications for diabetic retinopathy. *Experimental Diabetes Research*.

[9] Harijith A., Ebenezer D. L., Natarajan V. (2014). Reactive oxygen species at the crossroads of inflammasome and inflammation. *Frontiers in Physiology*.

[10] Lamkanfi M., Dixit V. M. (2012). Inflammasomes and their role in health and disease. *Annual Review of Cell and Developmental Biology*.

[11] Strowig T., Henao-Mejia J., Elinav E., Flavell R. (2012). Inflammasomes in health and disease. *Nature*.

[12] Paramel G. V., Sirsjö A., Fransén K. (2015). Role of genetic alterations in the NLRP3 and CARD8 genes in health and disease. *Mediators of Inflammation*.

[13] Aachoui Y., Sagulenko V., Miao E. A., Stacey K. J. (2013). Inflammasome-mediated pyroptotic and apoptotic cell death, and defense against infection. *Current Opinion Microbiology*.

[14] Schroder K., Tschopp J. (2010). The inflammasomes. *Cell*.

[15] Broz P. (2016). Inflammasomes: intracellular detection of extracellular bacteria. *Cell Research*.

[16] Broz P., Dixit V. M. (2016). Inflammasomes: mechanism of assembly, regulation and signalling. *Nature Reviews. Immunology*.

[17] Horng T. (2014). Calcium signaling and mitochondrial destabilization in the triggering of the NLRP3 inflammasome. *Trends in Immunology*.

[18] Ratsimandresy R. A., Dorfleutner A., Stehlik C. (2013). An update on PYRIN domain-containing pattern recognition receptors: from immunity to pathology. *Frontiers in Immunology*.

[19] Dinarello C. A. (2009). Immunological and inflammatory functions of the interleukin-1 family. *Annual Review of Immunology*.

[20] Chen Y., Smith M. R., Thirumalai K., Zychlinsky A. (1996). A bacterial invasin induces macrophage apoptosis by binding directly to ICE. *The EMBO Journal*.

[21] Yu J., Nagasu H., Murakami T. (2014). Inflammasome activation leads to caspase-1-dependent mitochondrial damage and block of mitophagy. *Proceedings of the National Academy of Sciences of the United States of America*.

[22] Danelishvili L., Bermudez L. E. (2013). Analysis of pyroptosis in bacterial infection. *Methods in Molecular Biology*.

[23] Chang W., Lin J., Dong J., Li D. (2013). Pyroptosis: an inflammatory cell death implicates in atherosclerosis. *Medical Hypotheses*.

[24] Li X., Du N., Zhang Q. (2014). MicroRNA-30d regulates cardiomyocyte pyroptosis by directly targeting foxo3a in diabetic cardiomyopathy. *Cell Death & Disease*.

[25] Tan M. S., Tan L., Jiang T. (2014). Amyloid-*β* induces NLRP1-dependent neuronal pyroptosis in models of Alzheimer’s disease. *Cell Death & Disease*.

[26] Takahashi M. (2013). NLRP3 in myocardial ischaemia-reperfusion injury: inflammasome-dependent or -independent rolein different cell types. *Cardiovascular Research*.

[27] Sandanger Ø., Ranheim T., Vinge L. E. (2013). A role for NLRP3 inflammasome in acute myocardial ischaemia-reperfusion injury? Reply. *Cardiovascular Research*.

[28] Sandanger Ø., Ranheim T., Vinge L. E. (2013). The NLRP3 inflammasome is up-regulated in cardiac fibroblasts and mediates myocardial ischaemia–reperfusion injury. *Cardiovascular Research*.

[29] Luo B., Li B., Wang W. (2014). NLRP3 gene silencing ameliorates diabetic cardiomyopathy in a type 2 diabetes rat model. *PLoS One*.

[30] Giordano A., Murano I., Mondini E. (2013). Obese adipocytes show ultrastructural features of stressed cells and die of pyroptosis. *Journal of Lipid Research*.

[31] Liu Y., Lian K., Zhang L. (2014). TXNIP mediates NLRP3 inflammasome activation in cardiac microvascular endothelial cells as a novel mechanism in myocardial ischemia/reperfusion injury. *Basic Research in Cardiology*.

[32] Zhang X., Zhang J. H., Chen X. Y. (2015). Reactive oxygen species-induced TXNIP drives fructose-mediated hepatic inflammation and lipid accumulation through NLRP3 inflammasome activation. *Antioxidants & Redox Signaling*.

[33] Xue R., Lei S., Xia Z. Y. (2016). Selective inhibition of PTEN preserves ischaemic post-conditioning cardioprotection in STZ-induced type 1 diabetic rats: role of the PI3K/Akt and JAK2/STAT3 pathways. *Clinical Science*.

[34] Liu M., Zhou B., Xia Z. Y. (2013). Hyperglycemia-induced inhibition of DJ-1 expression compromised the effectiveness of ischemic postconditioning cardioprotection in rats. *Oxidative Medicine and Cellular Longevity*.

[35] Min W., Bin Z. W., Quan Z. B., Hui Z. J., Sheng F. G. (2009). The signal transduction pathway of PKC/NF-*κ*B/c-fos may be involved in the influence of high glucose on the cardiomyocytes of neonatal rats. *Cardiovascular Diabetology*.

[36] Yang C. M., Lee I. T., Hsu R. C., Chi P. L., Hsiao L. D. (2013). NADPH oxidase/ROS-dependent PYK2 activation is involved in TNF-*α*-induced matrix metalloproteinase-9 expression in rat heart-derived H9c2 cells. *Toxicology and Applied Pharmacology*.

[37] Kim Y. S., Kim J. S., Kwon J. S. (2010). BAY 11-7082, a nuclear factor-*κ*B inhibitor, reduces inflammation and apoptosis in a rat cardiac ischemia-reperfusion injury model. *International Heart Journal*.

[38] Lu B., Wang B., Zhong S. (2016). N-n-Butyl haloperidol iodide ameliorates hypoxia/reoxygenation injury through modulating the LKB1/AMPK/ROS pathway in cardiac microvascular endothelial cells. *Oncotarget*.

[39] Zhang Y., Liao H., Zhong S. (2015). Effect of N-n-butyl haloperidol iodide on ROS/JNK/Egr-1 signaling in H9c2 cells after hypoxia/reoxygenation. *Scientific Reports*.

[40] Marim F. M., Franco M. M., Gomes M. T., Miraglia M. C., Giambartolomei G. H., Oliveira S. C. (2017). The role of NLRP3 and AIM2 in inflammasome activation during *Brucella abortus* infection. *Seminars in Immunopathology*.

[41] Abe J., Morrell C. (2016). Pyroptosis as a regulated form of necrosis: PI+/annexin V−/high caspase 1/low caspase 9 activity in cells = pyroptosis?. *Circulation Research*.

[42] Li H., Liu Z., Wang J. (2013). Susceptibility to myocardial ischemia reperfusion injury at early stage of type 1 diabetes in rats. *Cardiovascular Diabetology*.

[43] Li H., Bian Y., Zhang N. (2013). Intermedin protects against myocardial ischemia-reperfusion injury in diabetic rats. *Cardiovascular Diabetology*.

[44] Ghaboura N., Tamareille S., Ducluzeau P. H. (2011). Diabetes mellitus abrogates erythropoietin-induced cardioprotection against ischemic-reperfusion injury by alteration of the RISK/GSK-3β signaling. *Basic Research in Cardiology*.

[45] Arslan F., Smeets M. B., O'Neill L. A. (2010). Myocardial ischemia/reperfusion injury is mediated by leukocytic toll-like receptor-2 and reduced by systemic administration of a novel anti-toll-like receptor-2 antibody. *Circulation*.

[46] Fink S. L., Cookson B. T. (2005). Apoptosis, pyroptosis, and necrosis: mechanistic description of dead and dying eukaryotic cells. *Infection and Immunity*.

[47] Zheng Y., Gardner S. E., Clarke M. C. (2011). Cell death, damage-associated molecular patterns, and sterile inflammation in cardiovascular disease. *Arteriosclerosis, Thrombosis, and Vascular Biology*.

[48] Hu X., Ma R., Lu J. (2016). IL-23 promotes myocardial I/R injury by increasing the inflammatory responses and oxidative stress reactions. *Cellular Physiology and Biochemistry*.

[49] Steffens S., Montecucco F., Mach F. (2009). The inflammatory response as a target to reduce myocardial ischaemia and reperfusion injury. *Thrombosis and Haemostasis*.

[50] Takahashi M. (2014). NLRP3 inflammasome as a novel player in myocardial infarction. *International Heart Journal*.

[51] Sharma D., Kanneganti T. D. (2016). The cell biology of inflammasomes: mechanisms of inflammasome activation and regulation. *The Journal of Cell Biology*.

[52] Sutterwala F. S., Haasken S., Cassel S. L. (2014). Mechanism of NLRP3 inflammasome activation. *Annals of the New York Academy of Sciences*.

[53] Doitsh G., Galloway N. L., Geng X. (2014). Cell death by pyroptosis drives CD4 T-cell depletion in HIV-1 infection. *Nature*.

[54] Fink S. L., Cookson B. T. (2006). Caspase-1-dependent pore formation during pyroptosis leads to osmotic lysis of infected host macrophages. *Cellular Microbiology*.

[55] Lamkanfi M., Dixit V. M. (2010). Manipulation of host cell death pathways during microbial infections. *Cell Host & Microbe*.

[56] Gaidt M. M., Hornung V. (2016). Pore formation by GSDMD is the effector mechanism of pyroptosis. *The EMBO Journal*.

[57] Yang J. R., Yao F. H., Zhang J. G. (2014). Ischemia-reperfusion induces renal tubule pyroptosis via the CHOP-caspase-11 pathway. *American Journal of Physiology-Renal Physiology*.

[58] Kovarova M., Hesker P. R., Jania L. (2012). NLRP1 dependent pyroptosis leads to acute lung injury and morbidity in mice. *Journal of Immunology*.

[59] Juliana C., Fernandes-Alnemri T., Wu J. (2010). Anti-inflammatory compounds parthenolide and Bay 11-7082 are direct inhibitors of the inflammasome. *The Journal of Biological Chemistry*.

[60] Irrera N., Vaccaro M., Bitto A. (2017). BAY 11-7082 inhibits the NF-*κ*B and NLRP3 inflammasome pathways and protects against IMQ-induced psoriasis. *Clinical Science*.

